# A call to action for improving clinical outcomes in patients with asthma

**DOI:** 10.1038/s41533-020-00211-x

**Published:** 2020-12-04

**Authors:** Andrew McIvor, Alan Kaplan

**Affiliations:** 1 Firestone Institute for Respiratory Health, St. Joseph’s Healthcare and McMaster University, Hamilton, ON Canada; 2 University of Toronto, Toronto, ON Canada

**Keywords:** Asthma, Health policy

The management and treatment of asthma has undergone major changes since the inhalation of smoke generated from burning henbane in 1500 BC and *Datura stramonium* roots in the nineteenth century to alleviate asthma symptoms. From the use of anticholinergic alkaloids as first-line treatment, through the development of rapid-onset adrenergic bronchodilators for symptom relief, the use of inhaled corticosteroids (ICS) to treat the underlying lung inflammation, the re-introduction of anticholinergics in the 2000s and the emergence of targeted biologic therapies, advances in asthma treatment have led to significant improvements in asthma morbidity and mortality. However, the disease burden remains significant, with asthma affecting 339 million individuals worldwide in 2016 (ref. ^1^). Poor asthma control, exacerbations and even death continue to occur due to largely preventable factors, such as inappropriate prescriptions and/or inappropriate medication use. In Canada, total asthma costs are estimated to reach almost CAN$4.2 billion by 2030 (ref. ^2^). Evaluation of direct healthcare costs over a 4-year period in British Columbia revealed that medication costs contributed most to total costs, followed by physician’s visits and hospitalisations^3^.

The availability of pharmacologic therapies, such as ICS alone or in combination with long-acting β_2_-agonists (LABAs) and, more recently, targeted biologics, has helped lower exacerbation risk. Yet, asthma attacks persist, and optimal asthma management remains a challenge, especially within primary care. Indeed, although guidelines and policies have been formulated and revised to reflect the improvements in asthma management and the emergence of new treatments, support and resources to enable primary care physicians to adopt the changes into routine clinical practice are inadequate^4^. In parallel, asthma care and management is often neglected by healthcare policymakers, especially in developing countries, and local guidelines are slow to reflect global changes^1,5^. Lack of prioritisation at a local, national or international level is undoubtedly contributing to the persistent economic, health and personal burden of asthma.

## A new era in asthma care

Until recently, asthma care and management included inherent paradoxes and failure to treat asthma as a fundamentally inflammatory disease^6^. For example, previous and some current guidelines recommend(ed) the use of as-needed short-acting β_2_-agonists (SABAs) as the first step in asthma management and as the preferred symptom reliever. However, SABAs do not address the underlying airway inflammation responsible for symptoms^7,8^. This is a concern because of growing evidence that even mild asthma patients are at risk of severe exacerbations^9–13^. While there is some variation due to the different definitions of exacerbations used^9^, several studies, from randomised clinical trials (RCTs)^10,11^ to real-world survey^12^ and database^13^ analyses, have shown that approximately 6–22% of patients with mild asthma experience at least one exacerbation a year. Moreover, regular use of SABA alone is associated with safety risks and poor clinical outcomes^14^. In the US, in paediatric and adult patients with asthma, the use of ≥3 canisters per year and ≥2 SABA canisters per 6 months, respectively, was associated with an increased risk of exacerbations^14^.

However, for more than 30 years, as-needed SABA has been recommended as the preferred symptom reliever. Coupled with the natural tendency of patients to seek symptom relief, the combination of the immediate yet transient relief and the ‘as-needed’-use approach encouraged patients to treat just their symptoms with SABAs, ignoring the need for regular preventive medication. System barriers and policies affecting drug procurement and pricing also make it harder for patients to access preventive medication while making it easier to access reliever medication^5,15^. Lack of patient education, perceived inefficacy of ICS (as unlike SABAs, ICS have no immediate effect on breathlessness), fear of side effects from corticosteroids, inadequate health literacy and, in some cases, patient’s psychological dependence on rescue medication further contribute to SABA over-reliance^16–19^. Patients also have an incorrect perception of what asthma control means and frequently overestimate their level of control^9^. Automatic repeat prescriptions of SABA without clinical review and the lack of clear guidelines about the appropriate ratio of SABA/ICS further confound the issue. Consequently, there is widespread SABA over-reliance and ICS underuse^20^.

Evidence-based revised treatment recommendations now place ICS at the forefront of asthma treatment and no longer recommend SABA monotherapy^8^. The 2019 and, more recently, the 2020 Global Initiative for Asthma (GINA) strategy recommends ICS–formoterol—a combination of a low-dose ICS and a LABA with a fast onset of action—as the preferred as-needed reliever for adults and adolescents belonging to GINA treatment steps 1 and 2, and in steps 3–5, for patients prescribed ICS–formoterol maintenance therapy^8^. As-needed budesonide-formoterol is currently approved for mild asthma in Canada, Brazil, Russia, Australia and New Zealand based on data from two Phase III RCTs^10,11^, and is further substantiated by open-label pragmatic clinical trials^21,22^ as an effective and safe means of reducing severe exacerbations in comparison with as-needed SABA or maintenance budesonide plus as-needed SABA. Even in paediatric patients, the use of ICS is emphasised, with GINA recommending low-dose ICS whenever SABA is taken.

These changes represent a fundamental shift in recommended asthma treatment, making safety and the prevention of asthma exacerbations important goals, even in patients with mild asthma. However, to enable tangible improvements in asthma outcomes, changes in treatment recommendations need to be integrated into routine clinical practice in primary care where most asthma patients are managed.

Despite all the progress made, in some countries, access to even basic asthma treatment such as ICS is out of reach for most patients^1,5^. Although efforts have been made in recent years to improve respiratory outcomes through international agency policies on chronic/non-communicable diseases, more needs to be done, especially to improve access to asthma controller treatment in developing countries.

## Areas for improvement

The shift in treatment recommendations in favour of ICS–formoterol fixed combination heralds a change in the right direction as it represents tailoring of asthma management to patient needs by harnessing patients’ innate behaviours. Historically, strategies to improve patient adherence to regular anti-inflammatory therapy with either ICS or ICS–LABA have not always resulted in significant improvements either because they are too complex and/or they increase patient burden by being too labour intensive^23^. The recommendation of as-needed ICS–formoterol as the preferred reliever in mild asthma patients and in those treated with ICS–formoterol is likely to have a positive impact as it uses a patient’s natural relief-seeking behaviour (due to the fast-acting property of formoterol for symptom relief) to effect change without the need to change patient behaviour.

It is also increasingly evident that having asthma symptom control as the purpose of asthma treatment has not yielded desired results. Poor asthma control remains widespread across all severities^9^. Poor medication adherence, lack of a self-management asthma action plan, poor patient–physician communication and comorbidities can make asthma control challenging. Patients with well-controlled asthma or mild intermittent asthma also experience life-threatening exacerbations^9,13^. Thus, a paradigm shift towards population-based preventive care, rather than a predominant focus on symptom control, is needed.

Timely and accurate diagnosis of asthma and its phenotypes is also needed to identify individuals most in need of treatment. As per the GINA strategy, diagnosis of asthma is based on a history of characteristic respiratory symptoms and evidence of variable airflow limitation^8^. However, accurate diagnosis of asthma in primary care is hindered by many factors, including failure to use objective lung function tests, challenges associated with measuring lung function, development of late-onset asthma and different causes of observed symptoms^24,25^. The availability of specialised centres, which make available multiple specialists beyond pulmonologists, such as allergists and gastroenterologists, may help facilitate accurate diagnosis of more complicated patients, such as those who fail to achieve control, have comorbidities and/or have a severe disease; however, for the majority of patients, primary care can play an integral role in identifying ‘treatable traits’ (such as severity, airflow limitation, eosinophilic airway inflammation and atopy) and providing timely, appropriate treatment^24,26^.

**Fig. 1:**
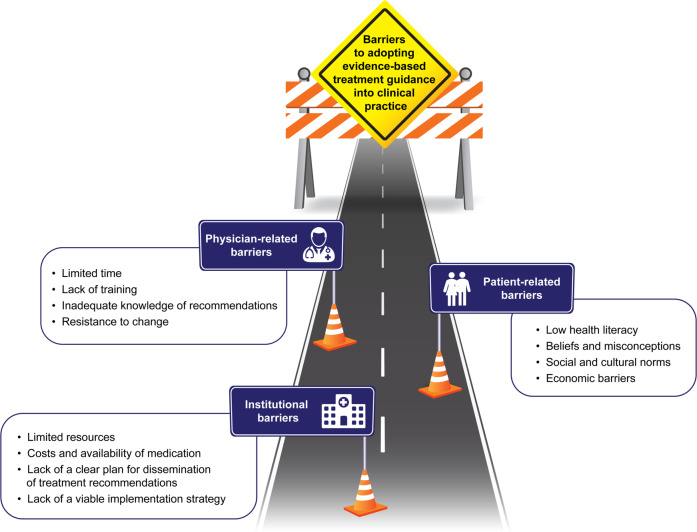
Institutional, physician- and patient-centred barriers to the adoption of evidence-based treatment guidance into clinical practice. Barriers to the adoption of evidence-based treatment guidance into clinical practice.

Even with a defined method of improvement, treatment practices will be slow to change. Overcoming institutional, physician- and patient-centred barriers^4,16,27^ (Fig. 1) is key to the adoption of these treatment changes into routine clinical practice. Institutional barriers include limited resources, outdated guidelines, lack of a clear plan for the dissemination of treatment recommendations and lack of viable implementation strategies such as targeted communication and educational initiatives. Physicians may lack the time or training needed, may have insufficient up-to-date knowledge of recommendations or may be resistant to change. Patient barriers include low health literacy, economic barriers, psychological dependence on SABAs and misconceptions, including a fear of side effects from corticosteroids. The wider implementation of evidence-based treatment recommendations may be hindered by incompatibility with country-specific standards of practice, including drug approval and coverage, as well as inertia within existing systems. These barriers must be surmounted to realise the complete benefits of adopting treatment changes. Evidence suggests that changes to healthcare systems will have an increased chance of success if driven by coordinated strategies at the national level^1,28^. In multiple countries, such as Finland, Poland and Brazil, national asthma programmes aimed at reducing asthma burden have been initiated and have resulted in improved asthma outcomes^28^. These programmes involve steps such as early diagnosis and introduction of anti-inflammatory treatment, promoting self-management, networking with physicians and, in Finland, legislation to decrease smoking and exposure to second-hand tobacco smoke. In Finland and Poland, critical to the programme’s success was the engagement of policymakers and commitment from physicians to alter asthma management practices in their clinics based on the programme. Moreover, patients, from non-governmental patient organisations were included as active partners towards deciding the implementation plan.

Thus, by working collaboratively, stakeholders—including policymakers, healthcare professionals, industry partners and patient groups—can drive policy-level changes to ensure system improvements that enable patients to be more easily identified by early diagnosis and, critically, make treatments available and accessible to patient populations across the world.

## A call to action

**Fig. 2:**
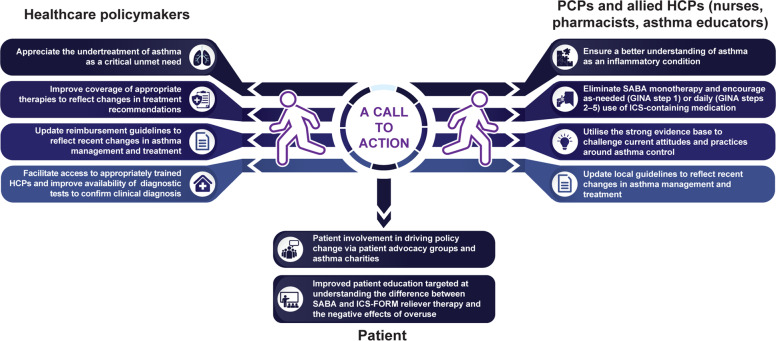
A call to action. GINA Global Initiative for Asthma, HCP healthcare professional, ICS inhaled corticosteroids, ICS-FORM inhaled corticosteroid–formoterol, PCP primary care physician, SABA short-acting β_2_-agonist.

Most asthma patients begin their treatment journey in a primary care setting. Most suffer from so-called mild asthma and are not likely to see an asthma specialist. Yet, even these patients are at risk of severe exacerbations and preventable asthma death. Thus, primary care physicians are in a unique position to drive change and improve asthma management. Appropriately trained allied healthcare professionals such as nurses, pharmacists and asthma educators are also key stakeholders in providing integrated care for chronic diseases such as asthma. Thus, recognising the growing asthma patient population and need for improved outcomes, this is a call to action for healthcare professionals and policymakers to shift to a new treatment strategy that harnesses patient behaviour rather than attempting to drastically modify it. For physicians and other healthcare professionals, key steps to realising the full benefit of this treatment change on patient outcomes include encouraging appropriate early ICS use, eliminating SABA monotherapy as per evidence-based recommendations, ensuring a better understanding of asthma as an inflammatory condition and active participation in adapting the GINA recommendations to local guidelines (Fig. 2). Healthcare policymakers need to appreciate the undertreatment of asthma as a critical unmet need and take measures to address this unmet burden. This can include creating a checklist of what appropriate care should include, followed by implementing policy changes aimed at improving healthcare coverage of appropriate therapies and updating reimbursement guidelines in line with recent treatment recommendations. Indeed, unlike SABA, ICS–formoterol may not be available or affordable in many developing countries. Thus, policy changes may be critical to improve accessibility to this anti-inflammatory reliever to prevent potential exacerbations and hospitalisations associated with SABA overuse. Additionally, policies aimed at increasing access to diagnostic tests (including alternatives to spirometry such as use of peak flow meters in resource-limited settings) to enable early diagnosis and timely treatment that is accessible to all patients are needed. In parallel, access to trained physicians needs to be improved. This can be achieved by including a payment for performance, virtual access for support to healthcare professionals if they have issues in care and integrated care systems to allow smoother information sharing and retrieval. These measures have the potential to improve clinical outcomes, such as reducing exacerbations and consequently, decreasing the burden on healthcare resources. However, to be truly effective, these steps will need to be accompanied by educational interventions targeted at patients and healthcare professionals and overall, improvement in access to asthma education in the community. Patient advocacy groups and asthma charities can also help drive this shift in asthma care by lobbying for policy changes and taking an active part in framing local guidelines. Thus, the achievement of this ambition will require action and collaboration among international agencies, policymakers, healthcare professionals, patient advocates and industry partners.

In conclusion, while SABAs have been the first step in asthma treatment for more than 30 years, the 2019 update in the GINA recommendations calls for a dramatic shift in the treatment of asthma^29^. Based on the safety risks associated with regular SABA use and over-reliance, GINA no longer endorses SABA monotherapy for patients with mild asthma but recommends ICS–formoterol as the preferred as-needed reliever for GINA steps 1 and 2, and in steps 3–5, for those patients prescribed ICS–formoterol maintenance therapy. Implementation of this change in the treatment and management of asthma has the potential to dramatically improve asthma clinical outcomes. However, for changes in evidence-based recommendations to translate into treatment changes in primary care, a concerted and collaborative effort will be required from primary care physicians, allied healthcare professionals, healthcare policymakers and patient groups.

### Reporting summary

Further information on research design is available in the Nature Research Reporting Summary linked to this article.

## Supplementary information

Reporting summary
